# Supplementary material for: T Cell Receptor-Directed Bispecific T Cell Engager Targeting MHC-Linked NY-ESO-1 for Tumor Immunotherapy
Source: Biomedicines. 2024 Apr 1;12(4):776. doi: 10.3390/biomedicines12040776 (PMC11048172; doi:10.3390/biomedicines12040776)
Supplement: Supplementary file 1 [file biomedicines-12-00776-s001.zip › Supplementary information.docx]

Supplementary information

TCR-directed bispecific T cell engager targeting MHC-linked NY-ESO-1 for tumor immunotherapy

Yiming Li ^1^, Wenbin Zhao ^1,2^, Ying Shen ^1,2^, Yingchun Xu ^1^, Shuqing Chen ^1,^* and Liqiang Pan ^1,^*

^1^ Institute of Drug Metabolism and Pharmaceutical Analysis, College of Pharmaceutical Sciences, Zhejiang University, Hangzhou 310058, China; yiming_li@zju.edu.cn (Y.L.); pharmacy_zwb@zju.edu.cn (W.Z.); shenying925@zju.edu.cn (Y.S.); ycxu66@163.com (Y.X.)

^2^ Zhejiang University Innovation Institute for Artificial Intelligence in Medicine, Engineering Research Center of Innovative Anticancer Drugs, Ministry of Education, Hangzhou 310018, China

***** Correspondence: chenshuqing@zju.edu.cn (S.C.); panliqiang@zju.edu.cn (L.P.)


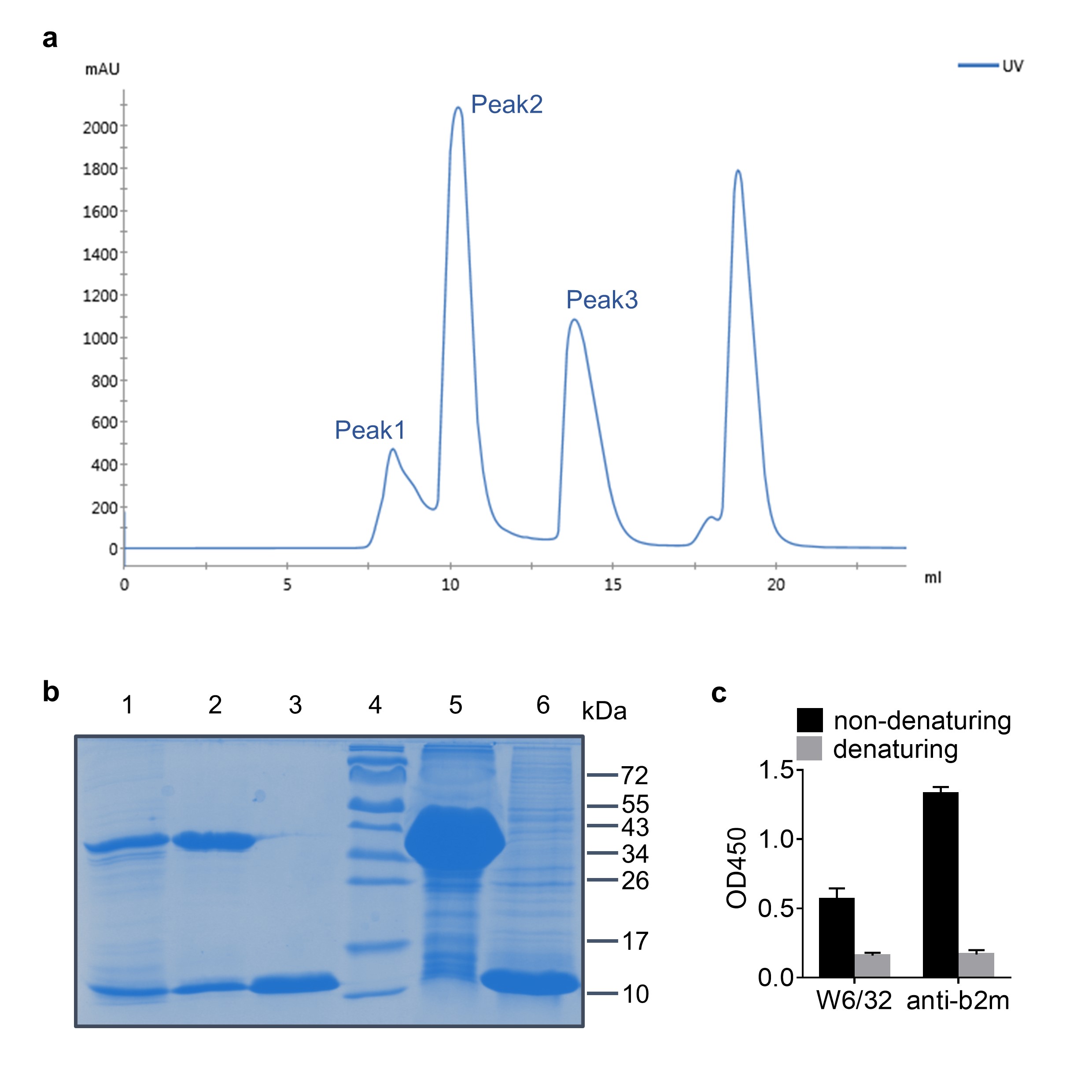


**Figure S1**. Characterization of homemade NY-ESO-1_157-165_/HLA-A*0201 monomer. (**a**) Purification of refolding peptide-MHC by molecular exclusion chromatography. The first three peaks were collected for the next characterization. (**b**) Reduced SDS-PAGE analysis of peaks from (a). Peak 2 showed two clear bands in the positions consistent with HLA-I and β2m. Lane 1, peak 1; Lane 2, peak 2; Lane 3, peak 3; Lane 4, prestained marker; Lane 5, HLA-I (~35 kDa) produced from E.coli; Lane 6, β2m (~11 kDa) produced from E.coli. (**c**) ELISA assay for testing the conformation of purified NY-ESO-1_157-165_/HLA-A*0201 monomer (peak 2) using W6/32 antibody and anti-β2m antibody.

Supplementary Method

*Generation of NY-ESO-1_157-165_/HLA-A*0201 monomer*

The peptide-MHC monomer was prepared by refolding as previously reported [1]. Briefly, β2m and the extracellular domain of HLA-I were expressed in *E.coli* and extracted from the inclusion body respectively. NY-ESO-1_157-165_ Peptide (SLLMWITQC), β2m, and HLA-I were added orderly in a folding reaction for 2-3 days. Then, the supernatant was concentrated and biotinylated, and the final peptide-MHC monomer was purified by molecular exclusion chromatography (Superdex^TM^ 75 increase 10/300 GL, GE Healthcare Life Sciences). The purified product was identified for the composition by reduced SDS-PAGE and conformation by ELISA using W6/32 antibody (Invitrogen) and anti-β2m antibody (B2M-01, Invitrogen) respectively with a common operation.

Reference

1. Denkberg, G.; Cohen, C.J.; Segal, D.; Kirkin, A.F.; Reiter, Y. Recombinant Human Single-Chain MHC-Peptide Complexes Made from E. Coli by in Vitro Refolding: Functional Single-Chain MHC-Peptide Complexes and Tetramers with Tumor Associated Antigens. *European Journal of Immunology* **2000**, *30*, 3522–3532, doi:10.1002/1521-4141(2000012)30:12<3522::AID-IMMU3522>3.0.CO;2-D.
